# Endoscopic ultrasound-guided gallbladder drainage in situs inversus totalis using a loop formation technique

**DOI:** 10.1055/a-2791-4426

**Published:** 2026-02-17

**Authors:** Sho Kitagawa, Narito Murakoshi, Naoki Shiga

**Affiliations:** 1 Department of Hepato-Biliary-Pancreatology, Sapporo Kosei General Hospital, Sapporo, Japan

Endoscopic retrograde cholangiopancreatography in patients with situs inversus totalis (SIT) is technically challenging because of the mirror-image anatomy, with ergonomic difficulties and scope instability often necessitating procedural modifications 1 2 3 . Endoscopic ultrasound (EUS) in patients with SIT also requires an accurate anatomical orientation within the mirror-image setting, with only a limited number of case reports published 2 4 5 . Herein, we present a case of EUS-guided gallbladder drainage (EUS-GBD) in a patient with SIT, highlighting the technical modifications employed to overcome these challenges.

**Fig. 1:**
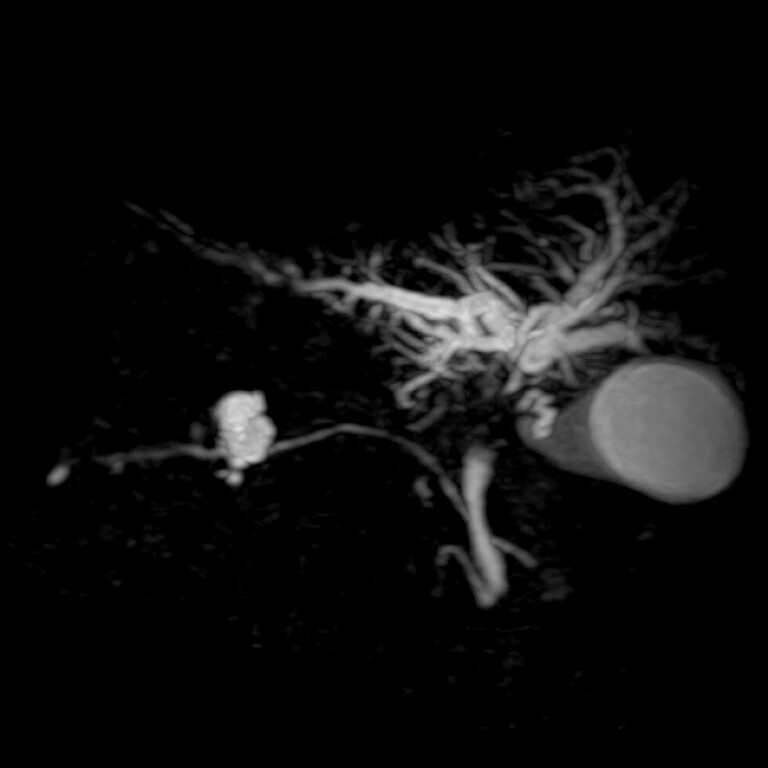
Magnetic resonance cholangiopancreatographic images showing hilar biliary strictures and cystic duct obstruction secondary to hilar cholangiocarcinoma in an 85-year-old woman with situs inversus totalis.

**Fig. 2:**
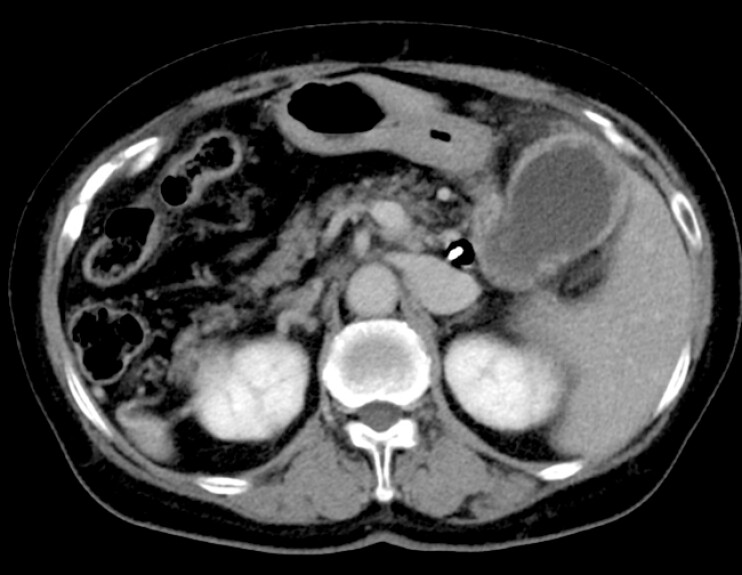
Computed tomographic images obtained before percutaneous transhepatic gallbladder drainage showing a distended gallbladder with wall thickening, consistent with acute cholecystitis.

**Fig. 3:**
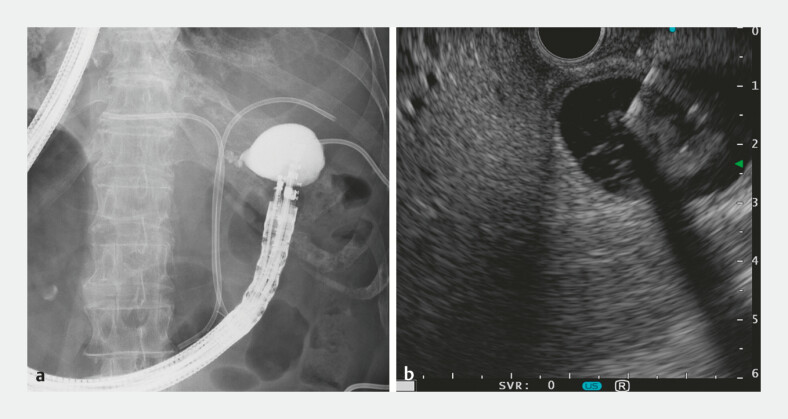
Endoscopic ultrasound-guided gallbladder drainage in situs inversus totalis. **a** Fluoroscopic and **b** endoscopic ultrasound images demonstrating the puncture of the gallbladder.

**Fig. 4:**
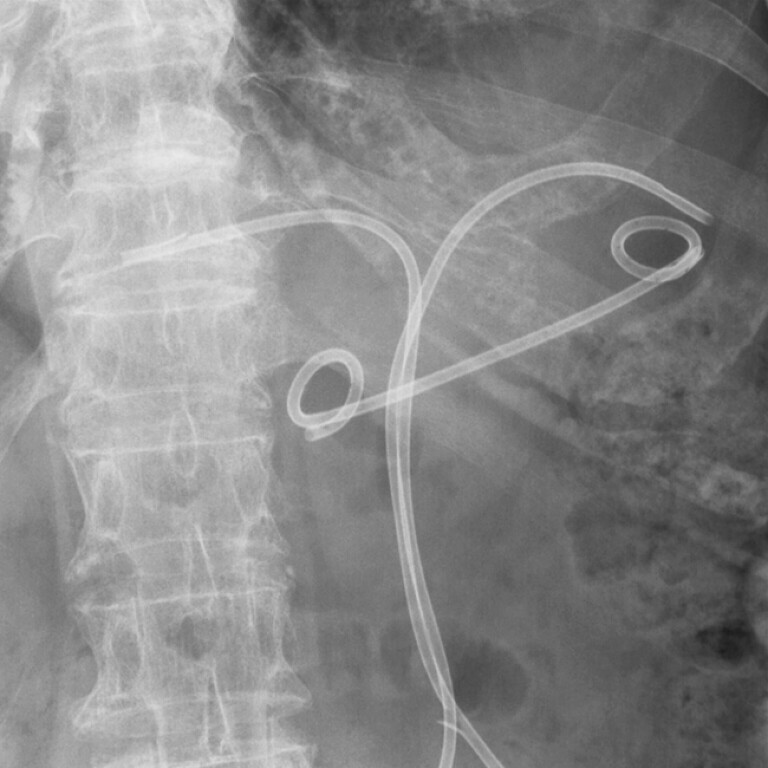
An abdominal radiograph showing the indwelling endoscopic ultrasound-guided gallbladder stent following the removal of the percutaneous drainage catheter.

An 85-year-old woman with SIT was referred for internalization of gallbladder drainage. She had previously undergone percutaneous transhepatic gallbladder drainage for cholecystitis caused by cystic duct obstruction secondary to hilar cholangiocarcinoma ( Fig. 1 and Fig. 2 ). EUS-GBD was performed with the patient in the prone position and the endoscopist standing on the right side of the table. Initially, the conventional mirror-image approach required the counter-clockwise rotation of the scope. However, this maneuver forced the endoscopist to extend the left hand away from the patient, resulting in an ergonomically compromised posture in which the endoscopist had to turn away from the patient. To address this issue, we deliberately rotated the scope counter-clockwise by 360 degrees to form a loop near the endoscopist’s hand. This technique allowed the EUS-GBD to be performed while maintaining a comfortable position facing the patient. In contrast to patients with normal anatomy, where the optimal visualization of the gallbladder from the duodenal bulb is typically achieved by slight scope withdrawal, the reversed anatomy in SIT renders the scope prone to slipping out, thereby precluding such maneuvers. Consequently, forward pressure had to be maintained throughout the procedure. Notably, loop formation contributed to scope stability, facilitating the successful placement of a 7Fr double-pigtail stent using the standard EUS-GBD technique ( Fig. 3 and Fig. 4 , Video 1 ).

Endoscopic ultrasound-guided gallbladder drainage in situs inversus totalis using a loop formation technique.Video 1

Endoscopy_UCTN_Code_TTT_1AS_2AH
